# Generalization of pain-related avoidance behavior based on de novo categorical knowledge

**DOI:** 10.1097/j.pain.0000000000002786

**Published:** 2022-09-20

**Authors:** Eveliina Glogan, Rena Gatzounis, Marc Patrick Bennett, Katharina Holthausen, Ann Meulders

**Affiliations:** a Centre for the Psychology of Learning and Experimental Psychopathology, KU Leuven, Leuven, Belgium; b Experimental Health Psychology, Maastricht University, Maastricht, the Netherlands; c Medical Research Council—Cognition and Brain Sciences Unit, University of Cambridge, Cambridge, United Kingdom; d Research Group Health Psychology, KU Leuven, Leuven, Belgium

**Keywords:** Avoidance behavior, Chronic pain, Operant conditioning, Generalization, Category learning

## Abstract

People with chronic pain often fear and avoid movements and activities that were never paired with pain. Safe movements may be avoided if they share some semantic relationship with an actual pain-associated movement. This study investigated whether pain-associated operant responses (movements) can become categorically associated with perceptually dissimilar responses, thus motivating avoidance of new classes of safe movements—a phenomenon known as category-based avoidance generalization. Using a robotic arm, 2 groups were trained to categorize arm movements in different ways. Subsequently, the groups learned through operant conditioning that an arm movement from one of the categories was paired with a high probability of pain, whereas the others were paired with either a medium probability of pain or no pain (acquisition phase). Self-reported pain-related fear and pain expectancy were collected as indices of fear learning. During a final generalization test phase, the movements categorically related to those from the acquisition phase were made available but in the absence of pain. Results showed that the generalization of outcome measures depended on the categorical connections between arm movements, ie, the groups avoided and feared the novel generalization movement categorically related to the pain-associated acquisition movement, depending on how they had previously learned to categorize the movements. This suggests that operant pain-related avoidance can generalize to safe behaviors, which are not perceptually, but categorically, similar to a pain-associated behavior. This form of pain-related avoidance generalization is problematic because category-based relations can be extremely wide reaching and idiosyncratic. Thus, category-based generalization of operant pain-related avoidance merits further investigation.

## Abstract 2

Supplemental Digital Content is Available in the Text.

In healthy participants, pain-associated movements were categorically associated with proprioceptively dissimilar pain-free movements, thus motivating unnecessary avoidance of the categorically associated, pain-free movements.

## 1. Introduction

The fear–avoidance model of chronic pain draws from psychological theories of learning and memory to explain the development and maintenance of chronic pain disability.^32,33^ This model proposes that pain-related fear is learned through *Pavlovian conditioning*, by which a stimulus (eg, a movement) paired with pain will begin to elicit fear in its own right.^20,21,30^ Subsequently, through *operant conditioning*, whereby one learns the consequences of their own behaviors,^32,33^ avoidance *responses* (eg, moving with reduced spinal motion^27^) perceived to omit pain will increase in frequency.^25^ If excessive, avoidance, in particular, may culminate into chronic disability by directly reducing physical activity and significantly interfering with daily functioning.^18^

Avoidance often spreads from a pain-associated movement to similar nonpainful/harmful movements—a phenomenon known as avoidance *generalization*.^10^ Avoidance can be generalized based on perceptual (eg, proprioceptive) similarities between responses (eg, back bending movements),^14,16^ but higher-order cognitive processes, like conceptual reasoning and verbal comprehension, can also be involved.^8,10^ For example, a person experiencing a shooting back pain in a specific yoga pose may begin to avoid this pose. Avoidance may subsequently generalize to other yoga poses, or yoga in general, even if perceptually these behaviors are different. If the person also categorizes “yoga” as similar to “exercise,” avoidance may be generalized to many different forms of exercise. Thus, individuals might build on their knowledge about a behavior's category membership when attempting to cope with pain.

Previous anxiety research^1,2,12^ demonstrated such category-based generalization of avoidance by training participants to group de novo stimuli (eg, nonsense words) such that novel categories emerged.^24^ Typically, following such category training, participants undergo Pavlovian fear conditioning. After this, they learn to avoid an aversive stimulus by employing an experimenter-instructed response (eg, pressing a computer button) when presented with a member from one of the categories. In a subsequent generalization test, presentations of other members from that category also elicit avoidance, despite never being paired with the aversive stimulus.^9^

In this procedure, the same avoidance response (button press) generalizes to differing *stimuli* (nonsense words). However, people with chronic pain often avoid different *behaviors* (ie, operant responses) with the goal of pain/harm reduction. Thus, in the context of pain, it may be helpful to focus on understanding how avoidance of one pain-associated *response* (eg, yoga) generalizes to other categorically associated *responses* (eg, exercise). To date, such category-based *response generalization*^25^ is largely unexplored.

This study investigated whether pain-related avoidance generalizes based on de novo categorical relationships between pain-relevant responses (arm movements). Categories were established that contained perceptually *similar* arm movements (response-congruent group) or perceptually *dissimilar* arm movements (response-incongruent group). Both groups then learned to avoid the same pain-associated arm movement (the shortest available arm movement) by deviating from it. Subsequently, the categorically associated generalization arm movements became available in the absence of pain. During the generalization test, we expected the response-congruent group to deviate more from the shortest available arm movement compared with the response-incongruent group who had learned to categorize the deviating pain-free arm movement from the acquisition phase, with the perceptually dissimilar, short arm movement from the generalization phase.

## 2. Method

### 2.1. Participants

Sixty-six, healthy, pain-free volunteers participated in this study. One participant was excluded prior to data analysis because they did not fulfill the inclusion criteria. Thus, 65 participants were included in the final analyses (48 women, mean ± SD [range] age = 25.82 ± 8.12 [18-65] years). The sample size was based on the generalization effect size from Glogan et al. (2021; *d* = 0.71), and an a priori power analysis was conducted in G*Power (independent-samples *t* test, 2 tailed, on mean avoidance behavior between groups during the first generalization block, *α* = 0.05, power = 0.80). Participants were randomly assigned to one of the 2 groups: response-congruent (n = 33) or response-incongruent (n = 32) group. Participants were recruited through the research participation system of the Maastricht University (Sona; Sona Systems, Nijmegen, the Netherlands), word of mouth, posters distributed around the university campus, and social media.

Exclusion criteria comprised the following: younger than 18/older than 65 years; pregnancy; left-handedness; chronic pain; acute pain in dominant shoulder/arm/elbow/wrist/hand; current/history of cardiovascular disease and/or psychiatric disorders; electronic implants (eg, pacemaker); uncorrected hearing and/or vision problems; analphabetism or diagnosed dyslexia; other serious medical conditions; and being advised to avoid stressful situations by one's general practitioner. Before the experiment, participants were informed that they could freely terminate participation at any time without any negative consequences, after which they completed an exclusion criteria checklist and an informed consent form. Participants were compensated either with €12.50 in gift vouchers or 1.5 course credit. The Ethics Review Committee Psychology and Neuroscience of the Maastricht University approved the experimental protocol (registration number: 185_09_11_2017_S12).

### 2.2. Apparatus

#### 2.2.1. HapticMaster

The HapticMaster (HM; Motekforce Link, Amsterdam, the Netherlands) is a 3-degree-of-freedom, admittance-controlled robotic arm. When operated by an external force, the robot reacts with a corresponding movement. By recording relative displacement from the starting position, the HM also provides an outcome measure to quantify each performed movement. For this study, the HM was programmed to allow movement along a two-dimensional, horizontal movement plane (depth = 0.36 m, radius = 0.40 m). The movement coordinates can also be used to conditionally trigger presentations of stimuli. This was used in the current experiment for delivering pain stimuli (see “*2.3. Pain stimulus*”).

#### 2.2.2. Software and hardware

The experiment was programmed in C#, using cross-platform game engine Unity 2019 (Unity Technologies, San Francisco, CA), and 3D graphics software, Blender 2.8 (Blender Foundation, Amsterdam, the Netherlands). The experimental script was run on a Windows 10 Enterprise (Microsoft Corporation, Redmond, WA) 64-bit Intel Core desktop computer (Intel Corporation, Santa Clara, CA) with 8 GB RAM and CPU: i7-7700 at 3.600 GHz. Communication between the computer and HM took place through a direct application programming interface connection. The experimental script was presented on a 40-inch LCD screen (Samsung UE40ES5500; Samsung Group, Seoul, South Korea).

### 2.3. Pain stimulus

The pain stimulus was a 2-ms square-wave electrocutaneous stimulus, delivered by a commercial constant current stimulator (DS7A; Digitimer, Welwyn Garden City, United Kingdom) through 2 reusable stainless steel surface disk electrodes (8-mm diameter with 30-mm spacing; Digitimer) filled with K-Y gel (Reckitt Benckiser, Slough, United Kingdom), attached to the triceps tendon of the participant's right arm. The intensity of the pain stimulus was calibrated individually for each participant, using a standard calibration procedure.^20^ This procedure consisted of the presentation of a series of electrical stimuli, starting at 1 mA and increasing in intensity in a stepwise manner.^15^ Participants were asked to reach an intensity that they would describe as “*significantly painful and demanding some effort to tolerate*,” approximating an “8” on a numeric rating scale ranging from 0 to 10. On this scale, 0 corresponded to “*I feel nothing*,” 1 to “*I feel something, but it is not unpleasant; it is only a sensation*,” 2 to “*the stimulus is not yet painful, but is beginning to be unpleasant*,” 3 to “*the stimulus is starting to be painful*,” and 10 to “*this is the worst pain I can imagine*.”

### 2.4. Experimental paradigm

**Figure 1.**
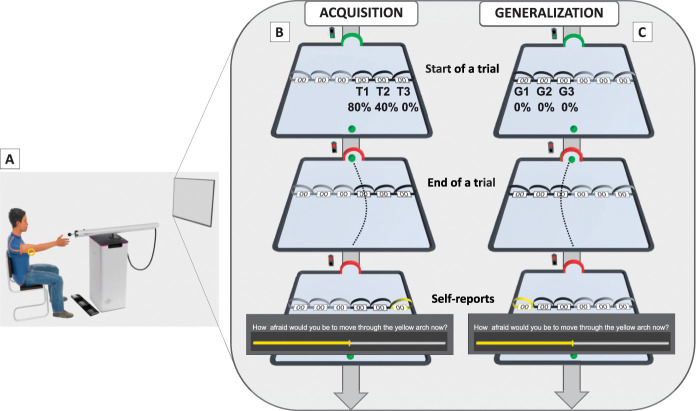
The experimental setup and a schematic overview of the experimental task. Panel (A): the participant is seated in front of the LCD screen, at reaching distance from the sensor of the HM. The electrodes for delivering pain stimuli are placed on the triceps tendon of the right arm (yellow circle), and the triple foot switch is used to give pain-related fear and pain-expectancy ratings. Panel (B): the acquisition phase of the current experiment, with T1-3 colored black and G1-3 colored grey. Panel (C): the generalization phase of the current experiment, with G1-3 colored black and T1-3 colored grey. Start of a trial*:* the target arch and traffic light turn green together with the auditory start tone. The green ball is situated at the center of the lower end of the movement plane. *Only for acquisition*: If the ball passes through T1, the pain stimulus is presented 80% of the time, and 40% of the time if moved through T2. End of a trial*:* the green ball passes through the target arch. The target arch and traffic light turn red, and the auditory stop tone is played. Self-report ratings*:* On some trials, a rating scale and question (pain expectancy/pain-related fear) appear on the screen. After both ratings have been given for all trajectories (T1-3 during acquisition and G1-3 during generalization), the HM automatically returns to its starting position, remains fixed for 3 seconds, after which the start signals are presented again, and the next trial starts. This figure is adapted with permission from 15. HM, HapticMaster.

The study employed an adaptation of the robotic arm-reaching paradigm, described elsewhere,^14,19^ which was extended to also include a category-formation phase.^24^ The participants' task was to move a “green ball” from a start location at the center of the side of the movement plane closest to the participant to a target location directly opposite, at the side farthest from the participant (see Fig. 1: panel B). Participants achieved this by operating the HM with their right (dominant) hand (see Fig. 1: panel A). Participants could visually track their movements, which were represented by the movement of the green ball, on screen in real time. The target location was visualized as a green arch, through which the green ball needed to be moved for a trial to be completed.

Participants could reach the target through 6 movement trajectories. The movement trajectories were indicated by 6 arches placed side by side, midway through the movement plane. Three movement trajectories (acquisition movement trajectories, T1-3) were located on the right side of the movement plane, and the other 3 movement trajectories were located on the left side of the movement plane (generalization movement trajectories, G1-3). T1 and G1 were the movement trajectories closest to the center of the movement plane and thus the shortest movement trajectories to the target (Fig. 1). T2 and G2 were placed next to T1 and G1, respectively, and thus included slightly more deviation to reach the target. Finally, T3 and G3 were placed at the far right and far left sides of the movement plane, respectively. Thus, they were the movement trajectories farthest away from the center and required the largest deviation to reach the target. In contrast to previous studies from our laboratory using the robotic arm-reaching paradigm, we did not manipulate the resistance paired with the movement trajectories in this study (for more information see Refs. 14, 19). This was done to prevent interference with category learning.

#### 2.4.1. Practice phase

The experiment started with a 6-trial practice phase, during which participants were acquainted with the HM and the task. The participant was presented with all movement trajectories. During the first 3 trials, only the movement trajectories on the right side of the movement plane (acquisition movement trajectories, T1-3) were available, and during the last 3 trials, only the movement trajectories on the left side of the movement plane (generalization movement trajectories, G1-3) were available. When movement trajectories were available, their trajectory arches were colored black, and when they were unavailable, their arches were colored grey (Fig. 1). On each trial, participants could freely choose between the available movement trajectories. The start of a trial was indicated by visual and auditory start signals: the target arch and a virtual traffic light located next to the target arch turned green, and a “start tone” was played. The trial ended when the green ball passed through the target arch. At this point, visual and auditory stop signals were presented: the target arch and the traffic light turned red, and a “stop tone” was played. Upon completing a trial, participants were asked to release the robot and wait for it to automatically return to its starting position. Participants were also able to practice providing self-reports of pain-related fear and pain expectancies using a triple foot switch (USB-3FS-2; Tokyo, Japan). A foot switch was used in order for self-reports to not interfere with the arm-reaching task. There were no pain stimuli during this phase.

#### 2.4.2. Matching-to-sample task

**Figure 2.**
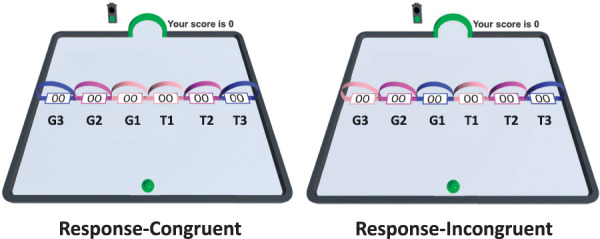
The movement trajectory categories of the response-congruent and response-incongruent groups. Note that, this figure has been created for visualization purposes, and the categories were never displayed to participants as such.

In the next phase, participants learned to group specific movement trajectories on the right side of the movement plane (acquisition movement trajectories, T1-3) with specific movement trajectories on the left side of the movement plane (generalization movement trajectories, G1-3). For the response-congruent group, the movement trajectory categories were T1 = G1 (the 2 shortest movements), T2 = G2 (the 2 middle movements), and T3 = G3 (the 2 longest movements). For the response-incongruent group, the categories were T1 = G3 (the short movement on the right side and the long movement on the left side of the movement plane), T2 = G2 (the 2 middle movements), and T3 = G1 (the long movement on the right side and the short movement on the left side of the movement plane) (Fig. 2). Thus, the response-congruent group learned to group the perceptually *similar* movement trajectories together (ie, short movements formed one category and long movements formed another), whereas the response-incongruent group learned to group perceptually *dissimilar* movement trajectories together (ie, short movements formed categories with long movements).

**Figure 3.**
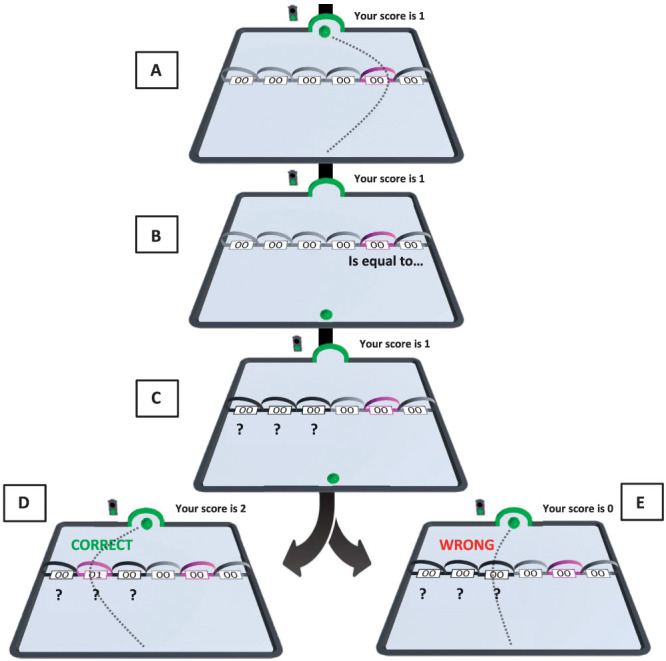
An example trial of the MTS task. (A) One of the acquisition trajectory arches (T1-3) is highlighted in its respective color (here, T2 in purple; ie, the sample movement). The highlighted movement is performed, after which (B) the message “Is equal to …” is presented. Subsequently, (C) question marks appear under the generalization trajectory arches. Simultaneously, the generalization movement trajectories (ie, the comparison movements) become available to be performed. (D) The participant performs one of the generalization movements; if the performed generalization movement is correctly matched with the previously highlighted and performed sample movement, the generalization trajectory arch is highlighted in the same color as the previously performed sample movement, the message “CORRECT” appears on the screen, and the counter increases by 1 unit. (E) If the movement trajectories are not correctly matched, the performed generalization movement trajectory arch is not highlighted, the message “WRONG” appears on the screen, and the counter is reset to 0. MTS, matching to sample.

During this phase, participants received feedback after each trial, indicating whether they had correctly matched the movement trajectories. The trial started with all 6 movement trajectories visible and colored grey. Following the start signals, the participant was instructed to perform a specific movement on the right side of the movement plane (ie, acquisition movement T1, T2, or T3). The to-be-performed movement (ie, the sample movement trajectory) was indicated by its trajectory arch being highlighted in a specific color. For T1, this was pink, for T2, it was purple, and for T3, it was blue. After the participant had performed the instructed movement, the message “Is equal to …” was presented on screen. Following this, the movement trajectories on the left side of the movement plane (ie, generalization movement trajectories, G1-3) became available (ie, their trajectory arches turned black) and question marks appeared below all of them, prompting the participant to perform a generalization movement of their choice (ie, the comparison movement trajectory). If the participant selected the *correct* movement trajectory, the following feedback was presented: an on-screen message saying, “CORRECT” in green letters, the chosen generalization trajectory arch being highlighted in the same color as the corresponding acquisition trajectory arch, and a counter at the top of the movement plane increasing by one unit (Fig. 3). If the participant selected an *incorrect* generalization trajectory, a written message saying, “WRONG” in red letters, was presented, the generalization trajectory arch remained black, and the counter was reset to 0. After this, a new trial with a new, randomly assigned sample movement trajectory began. That is, if a participant incorrectly matched the comparison movement trajectory to the sample movement trajectory on one trial of the matching-to-sample (MTS) task, the next trial did not necessarily involve the same 2 movement trajectories, ie, the same movement trajectory category. This phase ended when the participant had performed 21 correctly matched trials in a row (ie, acquisition criterion). Subsequently, category learning was tested. Participants were required to correctly match the previously trained pairs in the absence of the feedback. That is, even if participants correctly matched the pairs, no on-screen message was presented, the arch of the chosen generalization movement trajectory remained black, and the counter was no longer visible. In this way, we aimed to model real-life category learning, such as the process of learning to categorize a specific yoga pose in the category “yoga.” Twelve correctly matched trials in a row were required for this phase to be completed.

#### 2.4.3. Acquisition phase

The next phase was similar to the practice phase. However, only the movement trajectories on the right side of the movement plane (acquisition movement trajectories, T1-3) were available and thus colored black, and participants could freely choose between them. Furthermore, pain stimuli were now presented according to the experimental movement–pain contingencies. That is, choosing the shortest acquisition movement trajectory (T1) was paired with an 80% chance of the participant receiving a pain stimulus, the middle one (T2) was paired with a 40% chance, and the longest (T3) was never paired with the pain stimulus. Thus, participants could partly or completely avoid the pain stimulus by choosing T2 or T3, respectively. The pain stimulus was presented at two-third of movement completion. In this way, we aimed to model the emergence of real-life avoidance following a painful event, such as experiencing pain in a yoga pose and subsequently avoiding that specific pose. The generalization trajectory arches on the left side of the movement plane were also visible but colored grey to signal their unavailability. The acquisition phase comprised 3 blocks of 12 trials. On 2 fixed, predetermined trials of each block, self-report questions (see “*2.5. Primary outcome measures*”) were presented.

#### 2.4.4. Generalization phase

This phase was similar to the acquisition phase except that (1) only the movement trajectories on the left side of the movement plane (generalization movement trajectories, G1-3) were available and colored black, whereas T1-3 (on the right side of the movement plane) were now unavailable and colored grey, and (2) no pain stimuli were presented. Thus, we aimed to test if pain, paired with one activity (eg, a specific yoga pose), would also be associated with a categorically similar activity (eg, other yoga poses), and whether this association would evoke fear and avoidance of that pain-free, yet categorically similar, activity as well. The generalization phase consisted of 2 blocks of 12 trials. Similar to the acquisition phase, self-reports were collected on 2 fixed, predetermined trials per block.

### 2.5. Coprimary outcome measures

#### 2.5.1. Avoidance behavior

Avoidance behavior was operationalized as the maximal horizontal deviation from the shortest possible movement trajectory within the available side of the movement plane, per trial. That is, because the shortest movement on the right side of the movement plane (acquisition movement trajectory, T1) was paired with the highest probability of pain, deviation from this movement was interpreted as avoidance behavior.^4,19^ The possible range of avoidance was 0 to 20 cm. This information was extracted from the coordinates of each performed movement, automatically logged by the HM.

#### 2.5.2. Self-reports: pain expectancy and pain-related fear

Self-reports of fear and expectancy of pain related to each of the available movement trajectories were recorded on predetermined trials during the acquisition and generalization phases. Fear is a common emotional response to pain and often (but not always) motivates avoidance behavior.^18^ Pain expectancies measure people's beliefs about whether pain will be present and can thus be interpreted as a measure of contingency awareness and threat appraisal.^3^ Questions were presented on screen using a visual analogue scale (VAS) ranging from 0 to 100 (0 = “not at all” and 100 = “very much”) and answered using the triple foot switch. To indicate which movement trajectory the question referred to, the corresponding trajectory arch was highlighted in yellow. Participants answered the following questions *“To what extent do you expect an electrical stimulus when moving through the yellow arch?”* (ie, pain-expectancy) and “*How afraid are you to move through the yellow arch?”* (ie, pain-related fear).

### 2.6. Secondary outcome measures

#### 2.6.1. Trajectory choice

Frequencies of choices of all movement trajectories were calculated. This information was automatically logged by the HM.

### 2.7. Data analysis overview

The hypotheses and analysis plan were preregistered on Open Science Framework (https://osf.io/wtypx). During the acquisition phase, we expected self-reports to follow the pattern T1 > T2 > T3 in both groups, with the crucial effect of interest being between the shortest and longest movement trajectories available during this phase, T1 (80% chance of pain) > T3 (0% chance of pain) (ie, acquisition of fear and pain expectancies), given that T2 was ambiguous (40% chance of pain). Furthermore, we expected maximal deviations to be larger during the last acquisition block (ACQ3), compared with the first acquisition block (ACQ1) in both groups, indicating acquisition of avoidance.^12,15^

During the generalization phase, we expected self-reports to follow the pattern G1 > G2 > G3, in the response-congruent group and the opposite pattern (G3 > G2 > G1) to emerge in the response-incongruent group (again with the main interest being between the shortest and longest available movements during this phase, G1 and G3), indicating generalization of fear and pain expectancies. Furthermore, we expected maximal deviations to be larger in the response-congruent group compared with the response-incongruent group, given that the former were trained to categorize the longest movement trajectory on the right side of the movement plane (acquisition movement trajectory, T3) with the longest movement trajectory on the left side of the movement plane (generalization movement trajectory, G3), whereas the latter were trained to categorize the longest acquisition movement trajectory (T3) with the *shortest* generalization movement trajectory (G1). We were mainly interested in the first generalization block (GEN1), given that this phase did not include pain stimuli, potentially causing fear and avoidance to decrease throughout the phase.

To test for acquisition and generalization of self-reports and avoidance behavior, we performed a series of repeated-measures (RM) analyses of variance (ANOVAs) and follow-up contrasts to examine our a priori hypotheses. The α level was set at 0.05. For RM ANOVAs, Greenhouse–Geisser corrections were applied to correct for sphericity violations. Holm–Bonferroni corrected *P* values are reported for multiple comparisons. The indication of effect size $\eta_{p}^{2}$ is reported for significant ANOVA effects, and Cohen *d* for significant planned contrasts. Repeated-measures ANOVAs and planned contrasts were performed in RStudio (version 2021.09.2+382; RStudio, PBC, Boston, MA, 2009-2022), using R (version 4.1.2.; The R Foundation for Statistical Computing, 2021, Vienna, Austria), with package afex (version 1.0-1^23^). Note that we also preregistered proportional odds models on our trajectory choice data. The results from these analyses aligned with our main analyses. Thus, for the sake of brevity, we report the details and results of these analyses in Supplemental Digital Content 1 (available at http://links.lww.com/PAIN/B723).

## 3. Results

### 3.1. Demographics

We ran paired-samples *t*-tests to check for baseline group differences. There were no significant group differences in age (*t*(63) = 0.28, *P* = 0.78), physical intensity (mA) of the pain stimulus chosen during calibration (*t*(63) = 1.44, *P* = 0.16), or self-reported intensity of the pain stimulus (*t*(63) = 1.67, *P* = 0.10.

### 3.2. Acquisition

#### 3.2.1. Avoidance behavior

**Figure 4.**
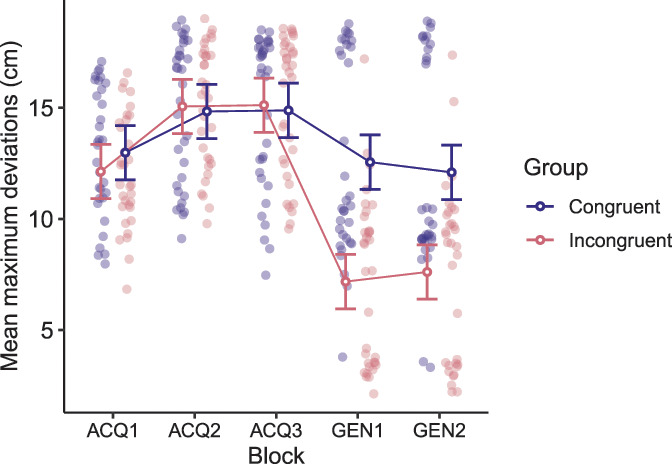
Mean maximum horizontal deviation from the shortest trajectory from the starting position to the target during the acquisition phase (ACQ1-3) and the generalization phase (GEN1-2), in the response-congruent (purple) and response-incongruent (pink) groups. Error bars represent 95% confidence intervals (CIs).

A 2 × 3 RM ANOVA on mean maximal deviations with group (response-congruent and response-incongruent) as the between-subject factor and acquisition block (ACQ1-3) as the within-subject factor yielded no significant 2-way interaction, F(1.82, 114.90) = 2.01, *P* = 0.14, but the expected significant main effect of block, F(1.82, 114.90) = 45.98, *P* < 0.001, $\eta_{p}^{2}$ = 0.42, suggested that avoidance behavior changed throughout the acquisition phase in both groups. Planned contrasts confirmed that both groups deviated more from the shortest possible movement trajectory on the right side of the movement plane (movement trajectory paired with the highest probability of pain, T1) by the end of the acquisition phase (ACQ3), compared with the beginning of this phase (ACQ1) (response-congruent: *t*(63) = 4.39, *P* = 0.0001, *d* = 0.60; response-incongruent: *t*(63) = 6.60, *P* < 0.0001, *d* = 1.03). This indicates successful acquisition of avoidance behavior (Fig. 4).

#### 3.2.2. Pain expectancy and pain-related fear

**Figure 5.**
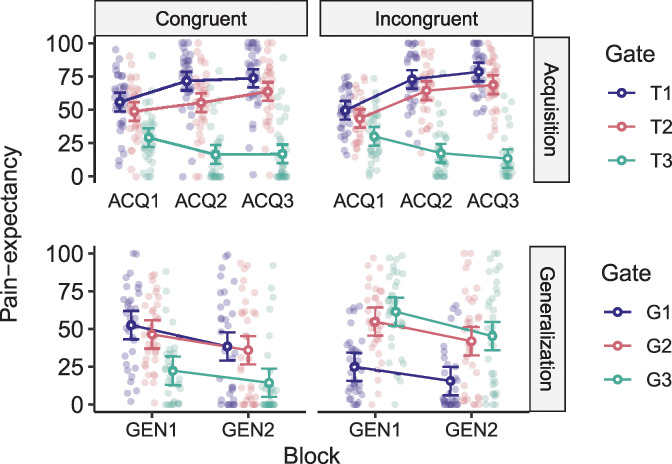
Mean pain expectancy toward the different movement trajectories in the response-congruent (left-hand panels) and response-incongruent (right-hand panels) groups during the acquisition (ACQ1-3; top panels) and generalization (GEN1-2; bottom panels) phases. Error bars represent 95% confidence intervals (CIs).

We ran a 2 × 3 × 3 RM ANOVA with group (response-congruent and response-incongruent) as the between-subject factor, and acquisition block (ACQ1-3) and trajectory (T1-3) as the within-subject factors, on mean *pain expectancies* during the acquisition phase. As expected, the 3-way interaction was not significant, F(3.69, 232.45) = 2.28, *P* = 0.07, but the block × trajectory interaction was F(3.69, 232.45) = 49.29, *P* < 0.001, $\eta_{p}^{2}$ = 0.44. This indicates that the differences in pain expectancies for the acquisition movement trajectories (trajectories on the right side of the movement plane) changed throughout the acquisition phase, similarly in both groups. Planned contrasts showed that, in line with our hypotheses, both groups reported significantly lower pain expectancy for the longest movement trajectory on the right side of the movement plane (pain-free acquisition movement trajectory, T3) compared with the shortest movement trajectory on the same side (movement trajectory paired with the highest probability of pain, T1), at the end of the acquisition phase (ACQ3) (response-congruent: *t*(63) = 9.89, *P* < 0.0001, *d* = 2.36; response-incongruent: *t*(63) = 11.20, *P* < 0.0001, *d* = 3.88) (Fig. 5: top panels).

**Figure 6.**
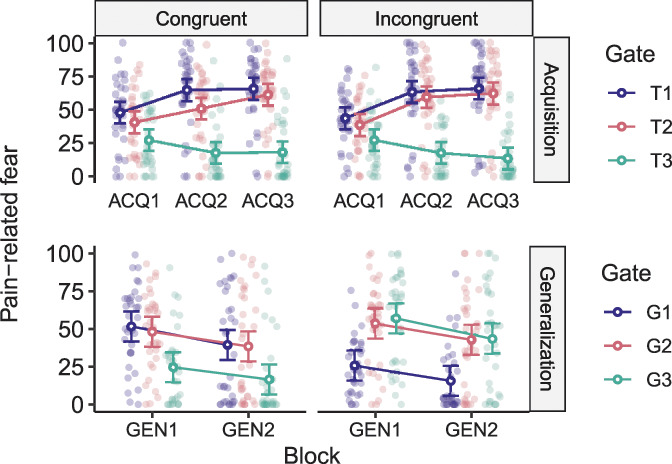
Mean pain-related fear toward the different movement trajectories in the response-congruent (left-hand panels) and response-incongruent (right-hand panels) groups during the acquisition (ACQ1-3; top panels) and generalization (GEN1-2; bottom panels) phases. Error bars represent 95% confidence intervals (CIs).

A similar RM ANOVA on *pain-related fear* ratings also revealed no significant 3-way interaction, F(3.03, 190.58) = 1.67, *P* = 0.18, but a significant block × trajectory interaction, F(3.03, 190.58) = 53.25, *P* < 0.001, $\eta_{p}^{2}$ = 0.46. Planned contrasts specified that similar to pain expectancies, both groups reported significantly lower fear for the longest, pain-free acquisition movement trajectory (T3) compared with the shortest one, paired with the highest probability of pain (T1) at ACQ3 (response-congruent: *t*(63) = 8.25, *P* < 0.0001, *d* = 1.96; response-incongruent: *t*(63) = 8.91, *P* < 0.0001, *d* = 2.16). Thus, both groups successfully learned the acquisition contingencies and showed differential fear reports reflecting these contingencies (Fig. 6: top panels).

### 3.3. Generalization

#### 3.3.1. Avoidance behavior

As expected, a 2 × 2 RM ANOVA on maximal deviations with group (response-congruent and response-incongruent) as the between-subject factor and block (GEN1-2) as the within-subject factor revealed a significant main effect of group, F(1, 63) = 23.48, *P* < 0.001, $\eta_{p}^{2}$ = 0.27, indicating general differences in mean maximal deviations between groups during this phase. Planned contrasts confirmed that deviations to the left were larger in the response-congruent group compared with the response-incongruent group, *t*(63) = 5.35, *P* < 0.0001, *d* = 1.33, at the beginning of the generalization phase (Fig. 4). During the category learning (MTS) task, the response-incongruent group learned to categorize the original avoidance response (long, pain-free movement on the right side of the movement plane, T3) with the *short* movement on the left side of the movement plane (generalization movement trajectory, G1). This group was therefore expected to “flip” their behavior between the acquisition phase and the generalization phase. That is, this group was expected to deviate less during the generalization phase. The response-congruent group learned to categorize the 2 short and 2 long movements together and were thus expected to continue deviating from G1 during the generalization phase. The finding of differing deviations between the groups thus suggests that both groups generalized avoidance based on the different categories they learned during the MTS task.

#### 3.3.2. Pain expectancy and pain-related fear

A 2 × 2 × 3 RM ANOVA with group (response-congruent and response-incongruent) as the between-subject factor and block (GEN1-2) and trajectory (G1-3) as within-subject factors, on *pain-expectancy* reports during the generalization phase, revealed the expected group x trajectory interaction, F(1.51, 95.43) = 39.96, *P* < 0.001, $\eta_{p}^{2}$ = 0.39. This indicates that the generalization movement trajectories (trajectories on the left side of the movement plane) evoked differing patterns of pain expectancies in the 2 groups. In line with our expectations, the response-congruent group (who learned to categorize the 2 short and 2 long movements together) expected pain more during the short movement trajectory on the left side of the movement plane (generalization movement trajectory, G1) compared with the long movement on the same side of the movement plane (generalization movement trajectory, G3), *t*(63) = 5.07, *P* < 0.0001, *d* = 1.22. In contrast, the response-incongruent group learned to categorize the long, pain-free movement on the right side of the movement plane (T3), with the short movement on the left side of the movement plane (G1). Thus, this group expected pain more during the long movement on the left side (G3) compared with the short movement on the same side (G1), *t*(63) = 6.17, *P* < 0.0001, *d* = 1.62, during the first generalization block (GEN1) (Fig. 5: bottom panels).

A similar RM ANOVA on *pain-related fear* reports revealed comparable effects to pain expectancies. There was a significant group x trajectory interaction, F(1.46, 92.06) = 32.63, *P* < 0.001, $\eta_{p}^{2}$ = 34, and planned contrasts showed significantly more fear for the short generalization movement trajectory (G1) compared with the long one (G3) in the response-congruent group, *t*(63) = 4.81, *P* < 0.0001, *d* = 1.04, and the opposite effect (G3 > G1) in the response-incongruent group, *t*(63) = 5.60, *P* < 0.0001, *d* = 1.25, at GEN1 (Fig. 6: bottom panels). These results indicate that participants generalized their pain expectancies and pain-related fear based on the de novo categories learned during the MTS task.

## 4. Discussion

In this study, we investigated whether pain-related avoidance generalizes based on (de novo) categorical relationships between pain-relevant responses (arm movements). In an MTS category-learning task, perceptually *similar* arm movements formed categories in one group (response-congruent). In the other (response-incongruent), those same arm movements formed categories of perceptually *dissimilar* arm movements. Subsequently, the groups learned to avoid the same pain-associated arm movement from one category (short movement on right side of movement plane, T1), by deviating from it (performing the long movement on the same side, T3). In a final generalization test, the arm movements on the left side of the movement plane, categorically associated with those directly paired with pain, became available but in the absence of any painful outcomes. During generalization, we expected the groups to behave according to the categories they had learned during the MTS task. That is, we expected the response-congruent group to deviate more from the shortest arm movement on the left (G1) compared with the response-incongruent group, given that the latter had learned to categorize the original avoidance response (long, pain-free movement on the right, T3) with that short movement on the left (G1).

Results supported these hypotheses; the response-congruent group expected pain more and were more afraid during the short movement on the left side (G1) compared with the long movement on this side (G3), whereas the opposite was true for the response-incongruent group (G3 > G1). Critically, both groups also generalized avoidance in line with the learned categories; the response-congruent group learned that the longest arm movement on the left side of the movement plane (G3) was associated with the initial avoidance response (long, pain-free movement on the right side, T3). This group thus showed larger deviations compared with the response-incongruent group, who had learned that the *shortest* movement on the left side (G1) was related to the original avoidance response (*long*, pain-free movement on the right side, T3). These findings indicate that avoidance of one painful action (eg, a specific yoga pose) can generalize categorically to movements, actions, or activities that a person associates with the initial painful action (eg, yoga as an activity or all exercise). This form of generalization is highly problematic, given that category-based relations can be extremely wide reaching and idiosyncratic because of their abstract nature and not being restricted by physical form. Category learning thus introduces another level of complexity to understanding the ways in which fear and avoidance behaviors can be acquired and maintained.^8^

The current results align with previous *anxiety* research.^1,11^ In these studies, participants performed an experimenter-instructed avoidance response (eg, pressing a computer button) during presentations of a stimulus (eg, nonsense word “AV3”) from one category, after learning that they could avoid an aversive stimulus (eg, a nonpainful electrical stimulus) during presentation of another member (eg, nonsense word “AV2”) of this category. However, AV3 had never been experienced with the electrical stimulus directly. Given that these previous studies did not use a *painful* stimulus, this study extends those findings to apply to *pain-related* avoidance as well. Furthermore, the current findings also extend previous ones by showing that not only (passive) Pavlovian stimuli but also (active) operant responses (eg, movements) can become categorically related to one another, resulting in avoidance of categorically similar, safe behaviors. This is especially relevant for chronic pain, in which fear and avoidance of *movement and activity* are some of the most important predictors of disability.^7,28,31^

Importantly, avoidance often comes at great personal costs to people with chronic pain (eg, disability, missing out on important life events, not being able to work).^22,26,13^ In previous studies from our laboratory on *perceptual* avoidance generalization, we have modeled these costs in the robotic arm-reaching paradigm by including a linear relationship between deviation and resistance from the HM robot.^14,16^ Thus, more physical effort of participants was required in those studies to perform the longest movements (avoidance responses, T3/G3), and these costs often attenuated avoidance generalization.^14,16^ We did not include costs in this study, to prevent interference with category learning. However, the current finding of (low-cost, category-based) avoidance generalization contrasts those previous findings of *attenuated* (costly, perceptual) avoidance generalization.^14,16^ This suggests that the minimized costs in this study may have enabled avoidance to generalize.

However, another study from the anxiety domain did demonstrate generalization of *costly, category-based* avoidance.^34^ In that study, participants underwent an avoidance test where, if they chose cards from a high-reward deck, they were presented with exemplars from a category (eg, animals), previously paired with a nonpainful electrical stimulus. Conversely, if participants chose cards from a low-reward deck, they were presented with exemplars from a category (eg, fruit) they had previously learned was safe. Participants in that study avoided the high-reward deck more than a control group for whom the avoidance test did not involve members from either of the fear-conditioned categories. Thus, avoidance generalized categorically at the cost of rewards.^34^ However, the previous robotic arm-reaching studies operationalized costs as physical effort,^14,16^ which may not be directly analogous to the comparatively indirect cost of missing a reward. Indeed, it is also possible that conceptual relationships are simply more salient than perceptual similarity and thus more conducive to avoidance generalization. Given these inconsistent methodologies and findings, the differing effects of high vs low costs, direct vs indirect costs, and perceptual vs category-based learning on avoidance generalization warrant further investigation.

The current findings partly align with a previous study^2^ where (non–pain-related) avoidance also generalized based on de novo categories. In that study, participants who learned a behavior competing with avoidance generalized avoidance only in the same context where it was first learned, whereas groups that did *not* learn any competing behaviors generalized avoidance also in different contexts. Thus, the behavior competing with avoidance seemed to attenuate its generalization to novel contexts,^2^ suggesting that reinforcing new categories of behaviors may be an effective strategy to reduce the generalization of avoidance also in pain. Rewards competing with avoidance have already been shown to decrease pain-related avoidance.^5,6^ Whether this approach applies to attenuating the category-based generalization of pain-related avoidance remains to be tested.

Some limitations deserve attention. First, we did not counterbalance the colors of the movement trajectory arches during the MTS task, possibly causing biased results. However, this is unlikely given that we did not use colors with any specific connotations (eg, green/red for approach/avoid). Second, in this experiment, we used only a low-cost avoidance response, meaning that there was no reason for participants *not* to avoid during the generalization phase. Although this may reduce the ecological validity of the results, it is important to note that people with chronic pain also use subtle and low-cost safety behaviors (eg, performing a feared movement with reduced spinal flexibility^27^ or keeping pain medication at hand at all times). Indeed, people may start with very low–cost avoidance to cope with their pain and subsequently habituate to the consequent safety and relief, thus going on to adopt more extreme solutions. Therefore, targeting low-cost avoidance at an early stage may be especially important in mitigating further functional decline.^29^ Low-cost avoidance behaviors may also be especially resistant to treatment,^17^ given that they do not hinder daily functioning (as much) and may go unnoticed by patients and therapists. Thus, we believe that these findings still bear clinical relevance. Given that this is the first experiment of its kind, we aimed to focus mainly on the category learning aspect—something which might have been disrupted by adding costs. A future study should aim to replicate the current findings with avoidance costs.

A further limitation is that for the response-congruent group, avoidance generalization was operationalized as longer deviations to reach the target (choice of G3), whereas for the response-incongruent group, avoidance generalization was operationalized as shorter deviations (choice of G1). Because G1 was the most direct movement trajectory to the target, it is difficult to exclude that the response-incongruent group was not simply behaving in the most energy-efficient way (rather than, or in addition to, generalizing). In line with this suggestion, post hoc *t*-tests on mean movement durations indicated that the response-incongruent group was significantly faster at performing the arm movements during the first generalization block, compared with the response-congruent group, *t*(64) = 3.16, *P* = 0.002. Unfortunately, whether participants perceived this as a cost cannot be reliably confirmed from the data we collected. Yet, given that self-reported pain-related fear and pain expectancies reflected the trained categories, as well as the observed avoidance behavior and our hypotheses, we do maintain that the most likely explanation for the group differences in avoidance behavior is the differing learning histories between the groups.

To conclude, the results of this study suggest that category knowledge can be engaged during the learning and generalization of operant pain-related avoidance. This form of generalization is highly problematic, given that category-based relations can be extremely wide reaching and idiosyncratic. Therefore, a better understanding of the mechanisms underlying category-based generalization of operant pain-related avoidance is needed–for which we believe that this study paves the way.

## Conflict of interest statement

The authors have no conflict of interest to declare.

This research has been presented at the Pain Science in Motion IV Congress 2022, taking place May 19-20 in Maastricht, the Netherlands.

## Appendix A. Supplemental digital content

Supplemental digital content associated with this article can be found online at http://links.lww.com/PAIN/B723.

## Supplementary Material
