# Supplementary material for: Generalization of pain-related avoidance behavior based on de novo categorical knowledge
Source: Pain. 2022 Sep 20;164(4):895–904. doi: 10.1097/j.pain.0000000000002786 (PMC10026827; doi:10.1097/j.pain.0000000000002786)
Supplement: Supplementary file 1 [file jop-164-0895-s001.pdf]

Generalization of pain-related avoidance behavior based on de novo categorical knowledge

Supplemental Digital Content 1: Description and results of proportional odds models

1. Data analysis overview

We ran proportional odds models to test (1) whether both groups were more likely to choose T3 than T1 during the final block of the acquisition phase, and (2) whether the Response-Congruent group was more likely to choose G3 compared to G1, and the Response-Incongruent group more likely to choose G1 compared to G3, during the first block of the generalization phase. We estimated two random intercept proportional odds models on trajectory choice data during the final acquisition block (Model 1) and the first generalization block (Model 2), with Trajectory Choice (Model 1: T1-3; Model 2: G1-3) as the categorical dependent variable, and Group (Response-Congruent, Response-Incongruent) as the predictor.

We ran these analyses using the R package brms (version 2.16.3;[1]). Brms fits Bayesian general linear multivariate multilevel models using the probabilistic programming language Stan[2], to estimate random effects proportional odds models. More specifically, the brm() function computes a sample of the posterior distribution of the model parameters, using Markov Chain Monte Carlo methods. Since the posterior sample allows computation of the distribution of any function of the model parameters, we used it to simulate the distribution of the probabilities of trajectory choices in each group[1]. Therefore, we were able to calculate the odds of one group (e.g. Response-Incongruent) choosing one movement trajectory (e.g. G1) over the others (e.g. G2 and G3), compared to the other group (e.g. Response-Congruent), as an odds ratio. In line with our *a priori* hypotheses, we also performed planned contrasts to examine the mean of the posterior

odds of both groups to choose T3 over T1, and of the Response-Incongruent group to choose G1 over G3, and of the Response-Congruent group to choose G3 over G1. If the odds equal 1, both movement trajectories have the same probability of being chosen. Odds greater than 1 indicate that the reference movement trajectory (e.g. G1) is more likely to be chosen than the comparison movement trajectory (e.g. G3). To account for the repeated-measures nature of our data, we included a random intercept for each participant in the models. The precision of the results obtained from our proportional odds models were estimated using 90% confidence intervals (CI). A regression coefficient was deemed statistically significant when the specific posterior credible intervals did not cross 0. Similarly, odds can be considered as statistically significant when the specific posterior credible intervals did not cross 1.

2. Results

2.1. Acquisition

The results of Model 1, with Trajectory Choice (T1-3) as the categorical dependent variable, and Group (Response-Congruent, Response-Incongruent) as the predictor, showed that participants in the Response-Incongruent group were 1.27 times more likely to choose T3 rather than T1 or T2 (or, T3 or T2 rather than T1), compared to participants in the Response-Congruent group, exp(.24), 90% CI [.34, 4.76] (see Fig. SDC1). Since an odds ratio of 1 indicates that the choice of a movement trajectory is equally likely to occur in both groups, this suggests that participants in the Response-Incongruent group, were only slightly more likely to choose T3 rather than T1 or T2 (or, T3 or T2 rather than T1), compared to participants in the Response-Congruent group, during the final acquisition block. One-sided follow-up contrasts (based on the mean of the

odds across participants) revealed that participants in the Response-Incongruent group were 35.01 times more likely to choose T3 than T1 during the final block of the acquisition phase, 90% CI [12.12, 76.23]. Similarly, the Response-Congruent group were 27.82 times more likely choose T3 than T1 during this block, 90% CI [9.86, 60.63].

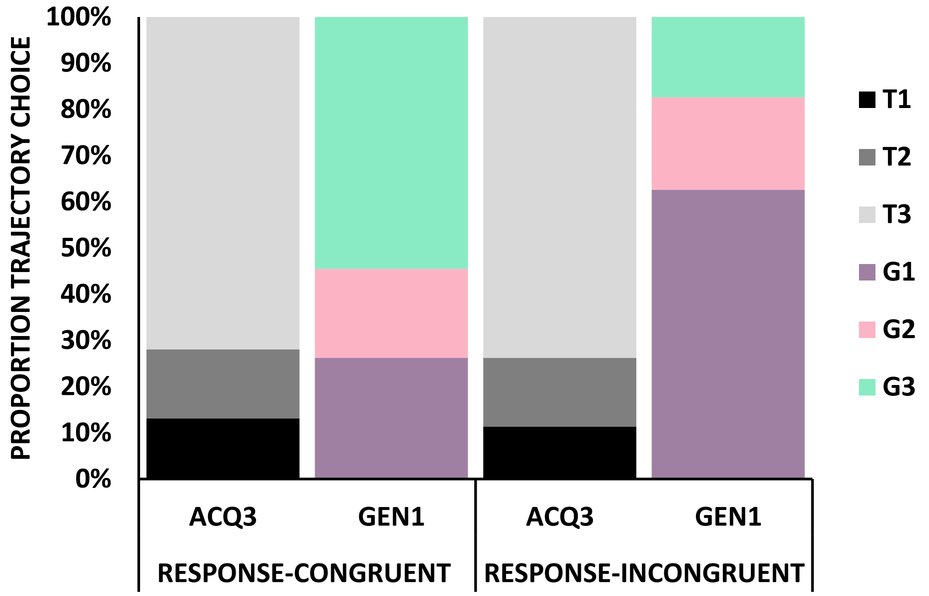

Fig. SDC1. Proportions of trajectory choices in the Response-Congruent and Response-Incongruent groups during the final acquisition block (ACQ3) and the first generalization block (GEN1).

2.2. Generalization

The results of Model 2, with Trajectory Choice (G1-3) as the categorical dependent variable, and Group (Response-Congruent, Response-Incongruent) as the predictor, showed that participants in the Response-Incongruent group were 54.05 times more likely to choose G1 rather than G3 or G2 (or, G3 or G2 rather than G1), compared to participants in the Response-Congruent group, exp(4.02), 90% CI [9.97, 379.93]. One-sided follow-up contrasts (similarly based on the

mean of the odds across participants) revealed that participants in the Response-Incongruent group were 23.54 times more likely to choose G1 than G3 during the first generalization block, 90% CI [5.80, 58.86]. In contrast, the Response-Congruent group were 11.50 times more likely to choose G3 than G1 during the first generalization block, 90% CI [2.53, 29.40].

In sum, the results of these analyses aligned with our hypotheses, and with the results from the RM ANOVAs on mean maximal deviations. That is, both groups respectively preferred the generalization movement trajectory (i.e. movement trajectory on the left side of the movement plane) that they had learned to categorize with the long, pain-free movement trajectory on the right side of the movement plane (T3; i.e. the avoidance response). In other words, both groups generalized avoidance based on the different categories they learned during the MTS task, indicating category-based avoidance generalization.

References

[1] Bürkner P-C. brms: An R Package for Bayesian Multilevel Models Using Stan. J Stat Softw 2017;80(1):28.

[2] Stan Development Team. Stan Modeling Language: User's Guide and Reference Manual, version 2.26.1. https://mc-stan.org, 2021.
